# Understanding the heterogeneous effect of microcredit access on agricultural technology adoption by rural farmers in Ethiopia: A meta-analysis

**DOI:** 10.1016/j.heliyon.2024.e35859

**Published:** 2024-08-06

**Authors:** Berhanu Kuma Shano, Samuel Semma Waje

**Affiliations:** Department of Agricultural Economics, Wolaita Sodo University, Wolaita Sodo, Ethiopia

**Keywords:** Ethiopia, Farmers, Microcredit access, Meta-analysis, Technology adoption

## Abstract

Though the Ethiopian economy is predominantly agriculture-based, the adoption of agricultural technologies has been very low. The results of a previous study had shown that microcredit access was one of the factors affecting the adoption of agricultural technology in Ethiopia. However, its effect has not yet been analyzed at the meta-level. Therefore, this study employed meta-analysis to understand the heterogeneous effect of microcredit access among farmers adopting agricultural technologies. We used subgroup analysis and meta-regression analysis to identify the heterogeneity level of credit access on technology adoption using the random-effects (RE) model. The study observed that there was a positive effect of microcredit access on agricultural technology adoption with a log odds ratio of 1.59. The subgroup analysis revealed a 93.2 % overall variation ($I^{2}$) with a p-value of 0.000, signifying a significant level of microcredit access within the between-groups heterogeneity of agricultural technology adoption studies conducted in Ethiopia. Notably, this was reflected by the adoption of improved livestock technologies, fertilizers, seed varieties, multiple agriculture, and irrigation technologies, with rates of heterogeneity of 94.9 %, 94.4 %, 94.3 %, 85 %, and 73.8 %, respectively, all with a p-value of 0.000. In addition, the meta-regression analysis results indicate that household experience, distance to the market, and income are significant moderators that affect the technology adoption decisions of farmers in rural Ethiopia. These findings suggest that policymakers should focus on improving the financial facilities and extension systems for rural farmers to enhance the adoption of agricultural technologies to increase production efficiency.

## 1 Introduction

The Ethiopian economy is mainly based on agriculture. However, the sector is struggling to meet the needs of rapidly growing population and reduce its dependence on food aid. Small-scale farmers play a main role in sustaining the sector [1,2]. In addition, Ethiopia's agricultural sector has the characteristics of traditional production systems, less cultivation land area (less than 2 ha), high vulnerability to shocks and food insecurity, limited availability of agricultural credit, a low level of market access, and a low level of new agricultural technology adoption [3]. However, 96 % of the country's food production rate is occupied by smallholder farmers, and their agricultural productivity performance is low [4,5].

In rural Ethiopia, various factors contribute to low productivity, including environmental influences such as climate change shocks, economic barriers such as financial constraints to purchase basic agricultural inputs and institutional factors [6]. However, it is important to note that the effect of technological barriers in increasing agricultural productivity, particularly the adoption of improved agricultural technologies, significantly impacts rural smallholder farmers’ productivity [[7], [8], [9]].

Low adoption of agricultural technologies at the household level can impact farmers' productivity. When farmers fail to adopt improved agricultural technologies, it decreases efficiency and impedes the potential for sustainable growth. Lacking access to these agricultural technologies, farmers are at risk of falling behind in an increasingly competitive market, where efficiency and output are crucial for success [10].

The Ethiopian government emphasized financial inclusion packages, particularly in stimulating rural microcredit provisions for smallholders, intending to increase production and crop yield rates. Notably, the allocation of funds to the agricultural sector has increased to more than ten percent of the public budget to scale up the agricultural extension and rural agricultural systems. One of the primary focuses of these efforts is to increase the production of major cereal crops such as maize, wheat, and barley [7]. This approach, in turn, helps to address food insecurity issues at the household level and leads to total economic growth in the country [9,11]. Despite these efforts, the new agricultural technologies, such as improved seed varieties, organic and non-organic fertilizers, and improved livestock technologies, have not been effectively implemented among smallholder farmers.

In addition, government-owned financial institutions (GOFIs) such as Commercial Banks, Development Banks, and private banks (PBs) cannot fill the financial gaps of smallholder farmers. The issue arises from the country's financial institutions' policies, i.e., the rules and regulations of formal financial institutions are not well designed to support rural households. Unsurprisingly, rural households have been asked about collateral requirements that they cannot afford. Similarly, microfinance institutions (MFIs) follow the same procedure. They are not coming to the poor people rather than serving the non-poor [5,12]. Moreover, weak financial institution outreach and liquidity challenges cause inefficiency in credit access limiting farmers' modern agricultural production [5,8,13].

The Ethiopian agricultural value addition increased from US $6.5 billion to US $20.02 billion between 2010 and 2020, while the country's grain output increased from 12 million metric tons to 23 million metric tons [7,14,15]. Notwithstanding these successes, the nation has had to deal with a sizable yield disparity. This yield gap is associated with a low level of agricultural technology access, knowledge, and use by smallholders [[16], [17], [18]]. Moreover, updated agricultural information is one of the critical factors for the yield gap [19]. For example, Abate et al. [7] reported that only 30–40 % of farmers utilize chemical fertilizers, and only 37–40 % of them apply technologies on their farms in Ethiopia.

Various theories have been applied to understand the concepts of adoption of agricultural technology in the field of agriculture since the pioneering work of Ryan and Gross [20] and Rogers [21]. These theories are categorized into three paradigms. The first paradigm involves diffusion theories such as diffusion of innovation theory (DIT) and technology lifecycle theory [21]. The theories focus on the relationships among technology, the environment, and organizations [22]. The opponents of this model, such as Feder [23], criticize the applicability of Rogers's categorization of adoption as “adoption” or “non-adoption” because it occurs on the continuum.

The second paradigm is the theory of user acceptance. The user acceptance theory includes the theory of reasoned action (TRA), the theory of planned behavior(TPB), the technology acceptance model(TAM), and its extensions such as TAM1, TAM2, and TAM3 [24]. This paradigm focuses on intrinsic factors such as knowledge, attitudes, and perceptions, along with extrinsic factors such as household characteristics and technological and environmental factors that can impact farmers’ decisions regarding technology adoption. The user acceptance paradigm also focuses on the utility maximization of a household; however, compared to the decision-making paradigm, the utility level may expand beyond only focusing on financial aspects.

The third paradigm involves decision-making theories, such as decision-making under uncertainty and risk management theories which focus on rational organizational and management interests [25]. This paradigm also focuses on the economic constraints that farmers propose for utility maximization when adopting technologies. However, this paradigm fails to capture the effects of the cultural aspect of an innovation [26]. The technology adoption theories explained above can provide some insights to practitioners and researchers on the factors affecting technology adoption dynamics in agriculture.

Furthermore, supply-side and demand-side issues are the two competing schools of thought that most significantly affect farmers' adoption decisions. On the demand side, the literature emphasizes the choice and game-theoretic models concerning farmers' risk perceptions, information gathering, training, accessibility to extension services, and social learning [27,28]. On the other hand, the adoption decision of new technology is influenced by changes in its supply [29]. Such changes or shifts in new technology adoption may result in a reduction in market price due to competition among technology producers. This study conceptually presents adoption theories to analyze the link between rural microcredit access and agricultural adoption decisions by interpreting predictor variables and adoption outcomes.

The methodology of meta-analysis has been a focus of literature in various research areas such as health [30], economics and econometrics [31,32], and ecology [33,34]. It provides the intellectual basis for systematic reviews, incorporates empirical data from several investigations, and seeks to provide a single pooled outcome [35]. In agriculture, some studies have applied systematic reviews and meta-analysis to their findings. For example, Schulz and Borner [36] synthesized farm-level adoption studies and explained their heterogeneity using meta-regression. Bastidav-Orrego et al. [37] employed a systematic review to evaluate agricultural policies using PRISMA. Ruzzante and Bilton [38] applied a meta-analysis of the empirical literature to analyze agricultural technology adoption in the developing world. Furthermore, Girma and Kuma [4] employed meta-analysis to analyze the effects of agricultural extension on market participation. However, there are scant meta-analysis studies in agricultural sector that specifically examine the link between microcredit accesses and adoption of agricultural technologies in sub-Saharan African countries. Therefore, this study applied meta-analysis to estimate the heterogeneous effect of microcredit access on the adoption of agricultural technologies in Ethiopia.

The previous literature on the adoption of rural agricultural technology in Ethiopia could be categorized fourfold. First, a large number of technology adoption studies have focused on improved seed varieties (ISVs) such as maize, wheat, barley, and sorghum [[39], [40], [41]]. Second, significant portion of studies have centered on adopting chemical and organic technologies [[42], [43], [44]]. Third, some studies have concentrated on the adoption of improved livestock technologies (ILTs) [45,46]. Finally, there have been studies focus on the adoption of improved irrigation technologies [[45], [46], [47]]. Moreover, several studies have been conducted on adopting multiple agricultural adoptions [6,[48], [49], [50], [51]]. These studies revealed that demographic and farm related, socioeconomic, and other factors in addition to microcredit access challenges using the cross-sectional data. Furthermore, there are studies that confirm access to microcredit significantly contributes to farmers’ decision whether to adopt new technology or not [5,7,43]. However, these studies have overlooked the diverse nature of microcredit access and its influence on the adoption agricultural technology.

To fill this literature gap, this study aims to conduct a Meta - analysis of published articles related to the adoption of agricultural technologies to answer the following questions.

(i) What is the level of heterogeneity of microcredit access among agricultural technology adoption studies in Ethiopia?
(ii) What are the key factors that influence the adoption of agricultural technology in Ethiopia?

Gaining a more profound insight into these issues could assist farmers' in encouraging and ensuring sustained adoption of agricultural technologies. This study contributes to the existing literature first by applying PRISMA (Preferred Reporting Items for Systematic Review and Meta-analysis) guidelines to collect articles and studies from different datasets. By adhering to this approach, the study aimed to ensure a comprehensive and methodologically rigorous approach to the collection and selection of relevant literature, thereby demonstrating a foundation for the agricultural technology adoption studies. Second, the study applied subgroup analysis using a forest plot diagram to estimate the heterogeneous effect of microcredit access on agricultural technology adoption using random effect model. By employing this method, our study not only provides a nuanced dynamics of microcredit influence but also advanced approach to analyze the connection between financial resources and technology adoption in agricultural sector. Third, the study employed meta-regression analysis to analyze the other determinants of agricultural technology adoption by farmers rather than access to microcredit. This is an important outcome to identify and quantitatively review the key factors affecting agricultural technology adoption at country level. Finally, the study applied Harbord's and Peter's regression-based tests to check publication biases among articles. Therefore, this distinguishes our paper, which provides more information to policymakers, research and financial institutions, and non-government organizations, emphasizing the importance of connection of farmers' access to microcredit and the adoption of agricultural technology in less developed countries.

## **2** Methods

**Fig. 1:**
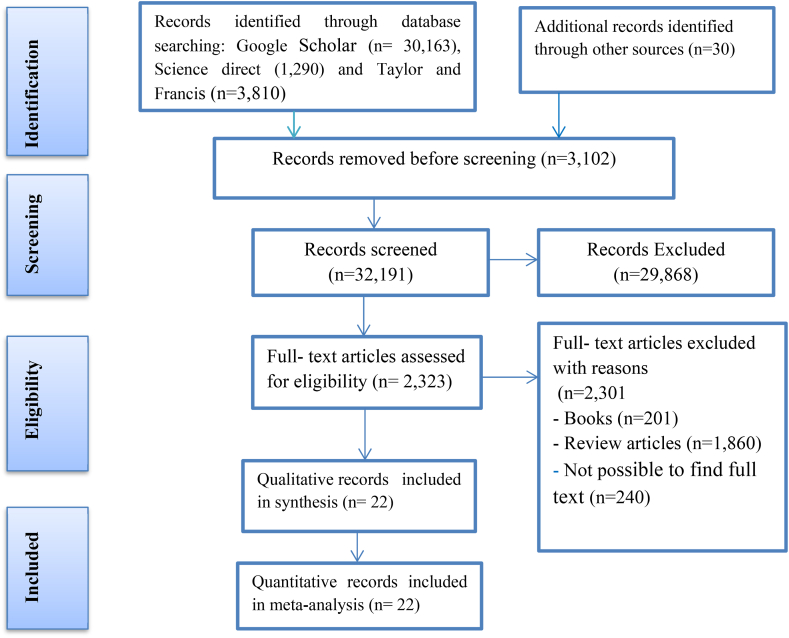
PRISMA 2009 flow diagram for selecting studies.

In the process of selecting studies, this study used PRISMA (Preferred Reporting Items for Systematic Review and Meta-analysis) approach, which consists of a 27-item checklist flow diagram based on Higgins et al. [52], Hutton et al. [33] and Liberati et al. [53] as shown in Fig. 1.

### 2.1 Browsing data

The study used Google Scholar, Science Direct, and Taylor and Francis databases to search for articles related to microcredit as a factor in adopting agricultural technology studies. These databases were selected due to their scope, which consists of a large portion of peer-reviewed studies and quality control standards. Articles were searched from the databases from February 2022 to May 2022. The combinations of the following keywords were used during the search. “Adoption” OR “technology adoption” OR “agricultural technology adoption” AND “microcredit” OR “credit access” OR “credit use” OR “agricultural credit service” AND “Ethiopia”.

### 2.2 Data extraction

The data extraction procedure was carried out to ensure data accuracy and reliability. The study used the year 1992 as the benchmark for the inclusion of studies. The rationale for selecting 1992 as the benchmark for our study was to capture the significant shift in the country's government structure and agricultural policy. Moreover, the year marked a turning point in the political and economic landscape, with far-reaching implications for the agricultural sector.

### 2.3 Study selection processes

The exclusion criteria for articles include duplication of research, studies without complete information, review articles, articles published outside of Ethiopia, lack of variables like access to credit, absence of the variable “adoption”, and use of agricultural credit. Using these exclusion criteria, a total of 35,271 articles were omitted, and 22 articles were used for quantitative meta-analysis, as shown in Table 1. Similarly, the method of selecting articles is presented in the PRISMA flow diagram in Fig. 1. This study used a total sample of 6123 household heads (Table 1).

**Table 1:** Effect size for binary outcomes.

Group	Yes	No	Size
Farmers who accessed microcredit	b	c	$n_{1}=b+c$
Farmers who have not accessed microcredit	d	e	$n_{2}=d+e$

In addition, we used agricultural technology adoption typologies (improved seed variety adoption, improved fertilizer adoption, improved livestock technology adoption, and improved irrigation technology adoption) for subgroup analysis. The study employed microcredit access (yes, no) as the intervention variable, and response variables (adopters and non-adopters) (see Table 1).

### 2.4 Data analysis

In this study, we specifically targeted articles that utilized a cross-sectional design for data collection and binary outcome models such as logit and probit. The study then proceeded to analyze the data for two-group comparisons of binary outcome variables using the random effect (RE) model in STATA. STATA measures the variables by considering the “event of interest” i.e. if a household head is access to microcredit, it is measured as “success” to mean “Yes”; otherwise, it is counted as “failure” to indicate “No” by the household head adopters and non-adopters of selected agricultural technology typology [30].

STATA software version 17 was used for the statistical analysis. The study first used the odds ratio to measure the effect of microcredit access on technology adoption and to indicate their linkages [54,55]. Second, the study employed the random effect (RE) model to assess the overall effect size and heterogeneity status within studies. One reason for using a random effect model rather than a fixed effect model is that the random effect model assumes that heterogeneity exists within the studies [54]. To check the status of heterogeneity among the typologies of rural agricultural technology studies, we first used subgroup analysis, and then a meta-regression analysis [4,54]. We used the Higgins et al. [52] classification for subgroup analysis to indicate the status of heterogeneity ($I^{2}$), which was calculated as ($I^{2}$ = 0, for no heterogeneity); ($I^{2}$ = 25 %, for low heterogeneity); ($I^{2}$ = 50 %, for medium heterogeneity); and ($I^{2}$ = 75 % and above, to indicate a high level of heterogeneity).

#### 2.4.1 Model specifications

The meta-analysis models have an important role in computing and interpreting the findings [55]. The models are two: fixed-effects and random-effects models. Their main difference is that the fixed-effects model assumes that the effect sizes of studies in Equation (1) are different and fixed. In contrast, the random-effects model assumes that the effect sizes of the studies in Equation (1) are different. The collected studies represent a random sample from more extensive population studies.

#### 2.4.2 Random-effects model

In this study, we employed the random-effects (RE) model. Applying a random-effects model is advisable for meta-analysis estimation [56,57]. Consider the following basic model:

1 $${\hat{\theta }}_{i}=\theta_{i}+\varepsilon_{i}\;i=1,2,\ldots ..N$$

where ${\hat{\theta }}_{i}$ is the estimated effect size, $\theta_{i}$ is the true effect size, $\varepsilon_{i}$ are sampling errors and $\varepsilon_{i}∼N\left(0,\sigma_{i}^{2}\right)$.N is independent agricultural technology adoption studies. Based on Equation (1),we have the following random-effects model in Equation (2):

2 $${\hat{\theta }}_{i}=\theta_{i}+\varepsilon_{i}=\theta +\mu_{i}+\varepsilon_{i}$$

$\mu_{i}$ is the error term and $\mu_{i}∼N\left(0,\tau^{2}\right)$ and $\varepsilon_{i}∼N\left(0,{\hat{\sigma }}_{i}^{2}\right)$.

#### 2.4.3 Odds ratio

The meta-analysis of binary data compares two groups: a treated group and a control group. Our focus was to measure the dummy responses indicated in the following 2×2 Table for the study presented in Table 1.

For farmers who accessed microcredit, $n_{1}$ is assumed to be fixed, b∼ binomial ($n_{1},\pi_{1}$), $\pi_{1}$ is the probability of accessing microcredit indicated by “yes” responses. Similarly, for farmers who did not access micro credit, $n_{2}$ is assumed to be fixed, d∼ binomial ($n_{2},\pi_{2}$), $\pi_{2}$ is the probability of accessing microcredit. The opposite works for the probability of failure (not accessing microcredit) indicated by “No” responses. Thus, the current study employed an odds ratio to estimate the effect sizes of the studies. The odds ratio computes an estimate of the log odds ratios. The odds ratio is the ratio of the odds of success (“yes” for our case) in the treatment group to the odds of success in control (“No” for our cases) (Equation (3)). The estimates of success probabilities like $\hat{\pi_{1}}=\frac{b}{n_{1}}$ for the treatment group and $\hat{\pi_{2}}=\frac{d}{n_{2}}$ for the control group.Hence, the odds ratio is calculated as follows:

3 OR=π1(1−π1)π2(1−π2)

The odds ratio estimated by$\hat{OR}=\frac{be}{dc}$.

The effect sizes of the meta-analysis use natural logarithms $\left(\hat{lnOR}\right)$ and computes using Peto's odd ratio and log odds ratio [55], which are expressed in Equations 4, 5) as follows:

4 $${\hat{OR}}^{Peto}=\exp\left\{\frac{b-E\left(b\right)}{Var\left(b\right)}\right\}$$

5 $$\ln\;\left({\hat{OR}}^{Peto}\right)=\frac{a-E\left(b\right)}{Var\left(b\right)}$$

#### 2.4.4 Meta-regression

Meta-regression explores the relationship between the study-effect sizes and the study-level covariates. Random-effects meta-regression analysis is important for estimating continuous variables. For the $i^{th}$ agricultural technology adoption study, let ${\hat{\theta }}_{i}$ denote the effect size, and ${\hat{\sigma }}_{i}^{2}$ be the variance of effect size, $\tau^{2}$ be a between-study component, and $X_{i}$ be a 1×a vector of moderators with the corresponding unknown a×1 coefficient vector β. The random effects meta-regression specified in Equation (6) as:

6 $${\hat{\theta }}_{i}=X_{i}\beta +\varepsilon_{i}^{*}=X_{i}\beta +\mu_{i}+\epsilon_{i},weighted\;by\;w_{i}^{*}=\frac{1}{{\hat{\sigma }}_{i}^{2}+\tau^{2}}$$

where $\varepsilon_{i}^{*}\;∼N\left(0,{\hat{\sigma }}_{i}^{2}+\tau^{2}\right)$, $\mu_{i}$ is the error term and $\epsilon_{i}$ is the error term for individual study effect. The other variables are explained above.

#### 2.4.5 Publication bias assessment tests

In meta-studies, regression-based tests and non-parametric rank-based tests are recommended for studying small-study effects [58]. The presence of asymmetry in small-study effects reflects the presence of publication bias. These tests aim to determine whether there is a statistically significant relation between effect sizes and their measure of precision [57]. Thus, we applied Harbord's and Peter's regression-based tests to check for small-study publishing bias in our study (see Table 4, Table 5).

## 3 Results

### 3.1 Articles identification

A total of 35,293 articles were identified through database searches, and 22 articles were used for quantitative meta-analysis, as presented in Table 2.

**Table 2:** Papers used for meta-analysis (all studies were conducted in Ethiopia).

Reference number	Name of authors'	Publication year	Typologies of Agricultural Technologies	Sample size	Study area(Regions)	Design of the study
[59]	Alemaw Teferi et al.	2020	Adoption of Improved Maize variety	300	Oromia	Cross-sectional
[60]	Leake & Adam	2015	Adoption of Improved wheat variety	160	Tigray	≫
[39]	Mesele Belay	2021	Adoption of Improved Sorghum variety	796	Amhara	≫
[61]	Fikadu et al.	2017	Adoption of improved livestock (Beehive)	268	Amhara	≫
[48]	Mihretie et al.	2022	Adoption of Improved Teff variety	224	Oromia	≫
[40]	Milkias and Muleta	2021	Adoption of Improved Barley variety	394	Oromia	≫
[62]	Milkias	2018	Adoption of Improved maize variety	150	Oromia	≫
[63]	Tarekegn and Assefa	2018	Adoption of improved livestock (Beehive)	360	Oromia	≫
[64]	Egge et al.	2012	Adoption of Improved Sorghum Variety	180	Somali	≫
[8]	Tesfaye et al.	2016	Adoption of improved Wheat Variety	120	Amhara	≫
[65]	Gecho and Punjabi	2011	Adoption of Maize	150	Oromia	≫
[41]	Yalew et al.	2020	Adoption of Improved Sorghum variety	306	Amhara	≫
[66]	Wondmeneh et al.	2014	Adoption of livestock technology (improved Poultry)	240	Oromia	≫
[67]	Zegeye and Assefa	2022	Adoption of Improved Wheat Variety	656	Amhara	≫
[49]	Zemarku et al.	2022	Livestock technology	199	South Ethiopia	≫
[50]	Tamirat	2021	Adoption of fertilizer	382	South Ethiopia	≫
[47]	Belete	2022	Adoption of fertilizer	192	Amhara	≫
[42]	Muluneh et al.	2022	Adoption of fertilizer	420	Amhara	≫
[68]	Gedafawu Abebe	2019	Improved fertilizer adoption	155	South Ethiopia	
[69]	Mohammed &Shallo	2020	Improved irrigation	140	South Ethiopia	≫
[51]	Agidew Abebe	2017	Improved irrigation	196	South Ethiopia	≫
[70]	Gebremeskel et al.	2018	Improved irrigation adoption	135	Tigray	≫
			Total sample size	6123

**Table 3:** Meta-regression results.

					Meta-regression	
					Number of obs	= 22
					Tau square	= 1.01
					I-squared residual	= 87.32 %
					Adj R-squared	= 66.04 %
					Prob > F	= 0.0010
Log(OR)	Coef.	Std. Err.	T	P > t	[95 % Conf.	Interval]
Age	−0.03010	0.0619421	−0.49	0.632	−0.1593091	0.0991089
Livestock(TLU)	−0.01121	0.1224067	−0.09	0.928	−0.2721185	0.249689
Distance to market	0.10128	0.0386503	2.62	0.017^b^	0.0203879	0.1821798
Experience in credit access	0.1307487	0.0407112	3.21	0.004^a^	0.0458267	0.2156707
Total annual income	0.0000439	0.0000189	−2.32	0.032^b^	−0.0000834	−4.26e-06
_cons	1.546928	0.4191211	3.69	0.001^a^	0.672657	2.4212

a p <1 %.

b p <5 %.

**Table 4:** Harbord's modified test for small-study effects.

Number of studies = 22					Root MSE = 3.948	
**Z/sqrt(V)**	Coef.	Std. err	T	p > t	[95 % conf. Interval]	
Sqrt(V)	−0.787902	1.085914	−0.73	0.477	−3.05308	1.477274
Bias	4.592522	3.888896	1.18	0.251	−3.519572	12.70462

Test of H_0_: no small study effect p = 0.251.

**Table 5:** Peter's test for small-study effects.

Number of studies = 22					Root MSE = 1.333	
Std eff	Coef.	Std. err	T	p > t	[95 % conf. Interval	
Bias	32.9223	145.5851	0.23	0.823	−270.7629	336.6075
Constant	0.5617253	0.7947207	0.600	0.360	−0.6899491	1.8134

Test of H_0_: no small study effects p = 0.823.

**Fig. 2:**
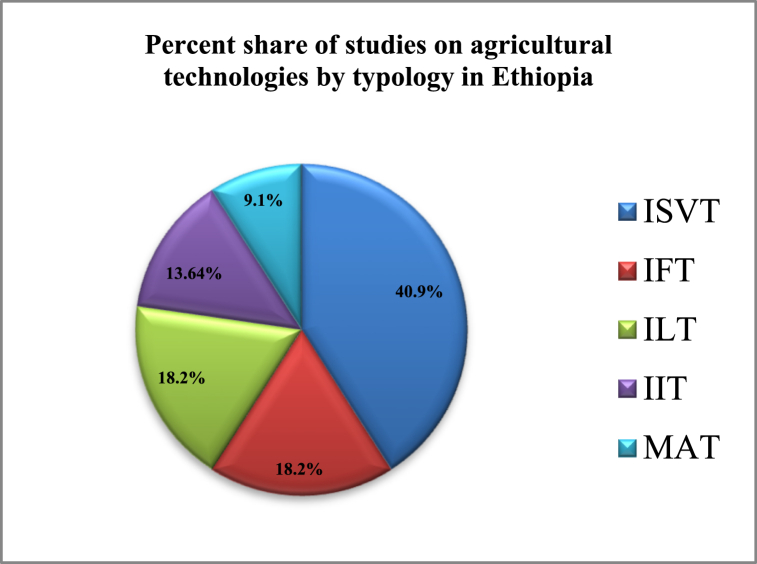
Percentage of agricultural technologies Note: ISVT = improved seed variety technology; IFT = improved fertilizer technology; ILT = improved livestock technology; IIT = improved irrigation technology; and MAT = multiple agricultural technologies.

Fig. 2 below presents the share of agricultural technologies by their typologies. The study found that large shares (40.9 %) of studies in Ethiopia were categorized under the improved agricultural seed technology (IST) typology; 18.2 % accounted for the adoption of both improved fertilizer technology (IFT) and improved livestock technologies (ILT). Moreover, improved irrigation accounts for 13.64 %, and multiple agricultural technologies account for 9.1 % of adoption.

**Fig. 3:**
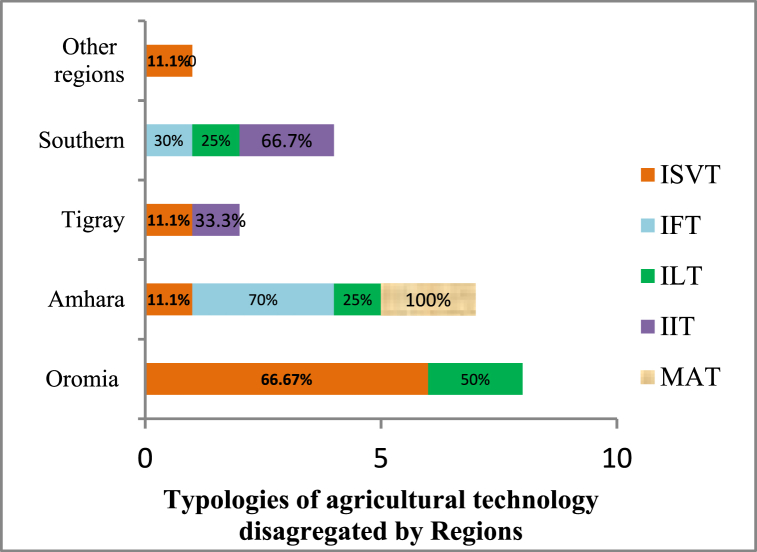
Agricultural technology typology by regions. Note: ISVT = improved seed variety technology; IFT = improved fertilizer technology; ILT = improved livestock technology; IIT = improved irrigation technology; and MAT = multiple agricultural technologies.

Similarly, Fig. 3 explains the adoption of agricultural technology typologies disaggregated by (study area) regions. The study found that improved seed variety adoption technologies are most common in the Oromia region (66.67 %), followed by Amhara (11.11 %), Tigray (11.11 %), and other regions. On the same explanation, 70 % of improved fertilizer adoption has mainly occupied the Amhara region and 30 % of the Southern regions. Improved livestock adoption practices are more common in the Oromia, Amhara, and South regions (refer to Fig. 3). Moreover, improved irrigation technology adoption is mostly practiced in the Southern and Tigray regions of Ethiopia (Fig. 3).

### 3.2 Microcredit access and agricultural technology adoption

The study used two types of meta-analysis methods to explore the status of heterogeneity of the adoption of agricultural technologies by their typologies. Following Higgins et al. [52] and Rice et al. [56], we first applied subgroup analysis to compare and analyze our study's binary outcome data (adopters vs. non-adopters and microcredit accessed or not accessed). Second, we used meta-regression analysis to analyze factors affecting agricultural technology adoption other than microcredit access by employing continuous variables (cofounders) to show heterogeneity within studies.

#### 3.2.1 Subgroup meta-analysis of the adoption of agricultural technologies

**Fig. 4:**
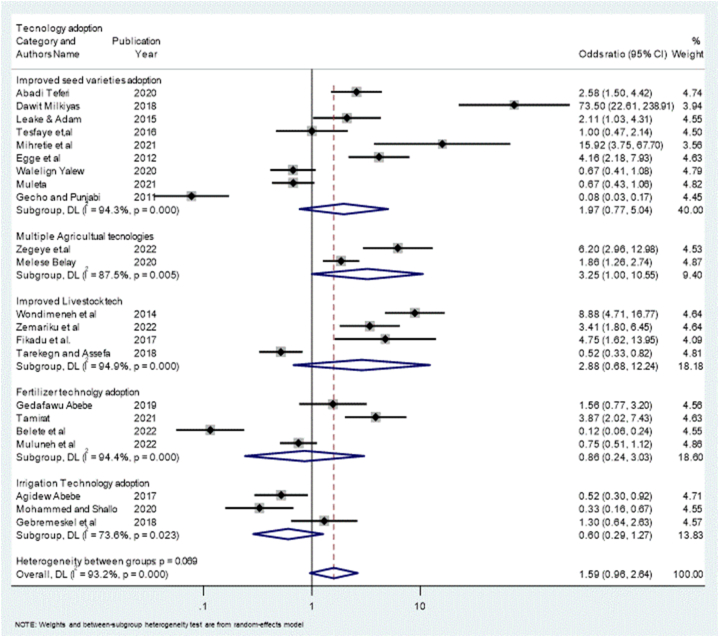
Forest plot displaying the random effect of meta-analysis, which describes the heterogeneous effect of microcredit access on adopting agricultural technology typology.

**Source**: own computation, 2022

Subgroup meta-analysis is a good way to address heterogeneity between studies [52]. Thus, the study categorized the technology adoption studies into five subgroups. These include the adoption of improved seed variety technology (ISVT), improved fertilizer technology (IFT), improved livestock technology (ILT), improved irrigation technology (IIT), and multiple agriculture technology (MAT). Fig. 4 presents the subgroups of agricultural technology typologies stratified by authors and year of publication, odds ratio, 95 % confidence interval, and percentages of weights.

Fig. 4 presents the sub-group analysis of different agricultural technologies contextualized with microcredit access, which has caused variation in technology adoption between adopters and non-adopters. The study analyzed the data by Meta-analysis pooling of Odds Ratios using the random-effects inverse-variance model, with DerSimonian-Laird estimate of ${\text{tau}}^{2}$ to test the effect size of each technology category and Cochran's Q statistics for heterogeneity identification. The forest-plot report revealed that the association between access to microcredit and the adoption of agricultural technology was represented by an overall odds ratio of 1.59. The $I^{2}$ (proportion of total variation in effect estimate due to between-study heterogeneity), which is represented by the heterogeneity of $I^{2}$ = 93.2 %, p = 0.000, indicating high heterogeneity in microcredit access and technology adoption of farmers in the study area. The results are in line with Girma [35], who analyzed the nexus between credit access and technology adoption in Ethiopia.

#### 3.2.2 Meta-regression results

Table 3 shows the meta-regression results of 22 studies conducted in Ethiopia. This study identified five moderators that are widely included in the above studies (see Table 1). The aim of including those variables in the meta-regression was to determine the relationships between the study effect in log OR and the selected covariates using a random effects model. The covariates (called moderators in the meta-analysis) included in the meta-regression model were the age of the households, tropical livestock unit (TLU), distance to the market, experience of using microcredit, and the total annual income of the farmers (Table 3).

Table 3 also shows that $I^{2}$ equals 87.32 % which suggests that there is high heterogeneity between-studies among agricultural technology adopters and non-adopters. In other words, the $R^{2}$ value which was equal to 66.04 % heterogeneity (variation) in the residuals was attributed to the between-study variations. In contrast, the remaining 33.96 % is attributed to within-studies variation in all five moderators. Moreover, the study showed that the combined result of all moderators with a p-value of 0.0010 suggests some correlation between at least one or two variables (moderators) and the treatment effect.

### 3.3 Publication bias and diagnostic tests

The study used Harbord's and Peter's modified tests to check for small-study publication bias among studies. The basic reason for this is that these tests are appropriate for binary data sets in meta-analysis [58] and to determine whether there is a statistically significant relationship between effect sizes and their measures of precision [52].

Table 4 presents the results of Harbord's test, which employs a weighted regression technique for small-study effects to investigate the link between effect sizes (log odds ratio) and precision. The model output shows that Harbord's modified test statistic has a t-value of −0.73 and a p-value of 0.251, indicating the presence of small study effects. Similarly, Table 5 illustrates the results of Peter's test for the small-study effect, which revealed that the calculated bias coefficient was 32.92 with a standard error of 145.58, a t-value of 0.23, and a p-value of 0.823 (Table 5). These results, show that there is no evidence to support the null hypothesis, which states that “H_o_ = no small-study effect”. This indicates that the technology adoption studies we conducted in Ethiopia have publication bias.

## 4 Discussions

This study examined the effect of microcredit access on agricultural technology adoption in Ethiopia by employing a meta-analysis approach. The study searched 35,293 articles through three datasets (see Fig. 1) using PRISMA to select 22 articles focused on Ethiopia.

### 4.1 Typologies and heterogeneity status of agricultural technology adoption

This study employed a sub-group meta-analysis approach, which categorized the agricultural technology adoption studies into five typologies: these include improved seed varieties technologies (ISVTs), improved fertilizers technologies (IFTs) adoption, improved livestock technologies (ILTs) adoption, improved irrigation technologies (IITs) adoption, and multiple agriculture (MAT) adoption (see Fig. 2, Fig. 3, Fig. 4).

In Ethiopia, a large share of agricultural technology adoption studies have focused on the adoption of improved seed varieties, followed by improved fertilizer and livestock adoption studies equivalently, and improved irrigation studies (see Fig. 2). This underscores the importance of improved agricultural technologies in the country's agricultural sector and their potential impact on agricultural productivity [26].

Similarly, the study identified these agricultural technology adoption studies disaggregated by geographical locations (see Fig. 3). As a result, the Amhara region accounts for 70 % of improved fertilizer studies, the Oromia region accounts for 60.67 % of improved seed variety adoption, and 66.77 % of improved irrigation technology adoption studies centered in the South region. Disaggregating studies into regional bases helps to highlight the distribution of adoption studies among regions that provide valuable contexts regarding the patterns seen in previous studies. It also provides a viewpoint to contextualize what typologies and geographic areas have received the most analytical attention. The findings of our study are in alignment with Amoussouhoui et al. [71], who examined adoption of digital agricultural technologies at global scale.

The heterogeneity status detected among agricultural technology studies in Ethiopia stems from various factors such as the diversity of technology types, sample size variation across studies, geographical locations or regions, and differences in the quality and quantity of agricultural technologies [7,8,35]. The subgroup analysis indicated the extent of the heterogeneity and revealed that improved livestock technology adoption exhibited $I^{2}$ of 94.9 % with p = 0.000, indicating a high level of variation among livestock technology studies. Similarly, improved fertilizer technology adoption demonstrated $I^{2}$ of 94.4 % with a p-value of 0.000, reflecting a substantial heterogeneity among the studies. Improved seed variety technology adoption had $I^{2}$ of 94.3% with p-value of 0.000. Furthermore, multiple agricultural technology adoption had $I^{2}$ of 87.5 % with a p-value of 0.000, whereas improved irrigation adoption had a $I^{2}$ value of 73.8% with a significance level of p = 0.000. This demonstrates that there is a high level of heterogeneity within these studies. These findings are in line with previous findings of [26,[72], [73], [74], [75]], who showed that improving the rural agricultural technologies of cereal crops has a greater effect on individual farmers’ income at a specific level and on the welfare of the farmers at a general level in Ethiopia.

### 4.2 The heterogeneous effect of microcredit access

The study examined the effect of microcredit access on agricultural technology adoption by employing the odds ratio to assess the effects using a random-effects model. The study's outcome indicated that microcredit access significantly contributed to technology adoption, yielding an odds ratio of 1.59 for adopters compared with non-adopters in Ethiopia. However, upon looking for the estimate of each typology of technology adoption studies, a notable heterogeneity was observed in the influence of microcredit access on technology adoption studies in Ethiopia (Fig. 4).

Specifically, the study revealed that microcredit access has a tangible effect on improved seed variety technology adoption (ISVT) among adopters, as evidenced by a log odds ratio of 1.97 compared to that of non–adopters. Similarly, compared with non-adopters, microcredit access impacted improved fertilizer technology (IFT) adopters by an odds ratio of 0.86. Moreover, compared with non-adopters, the effect of microcredit access was found to have a significant positive effect on the number of adopters of improved livestock technologies (ILT), with a log odds ratio of 2.88. According to Girma [35], agricultural credit access is a good way to tackle factors related to technology adoption in Ethiopia. Similarly, Mariyono et al. [76] identified the role of microcredit in sustaining household well-being in Indonesia.

Furthermore, rural microcredit access contributed adopters to adopting multiple agricultural technologies and improved irrigation technology, with odds ratios of 3.25 and 0.60 respectively as compared with non-adopters. This finding is in line with the findings of [46,77,^77^]. One explanation for this is that, even though many Ethiopian farmers are willing to adopt improved and modern livestock technologies, they face financial barriers such as a lack of well-organized microfinance institutions and services, which prevent them from using and adopting new and modern agriculture. This finding is in line with previous research of Kebebe [78] and Wonmeneh et al. [66], who recommended that farmers be capacitated with a rural credit service system to adopt livestock technologies.

### 4.3 Effects of other covariates

As shown in Table 2, the meta-regression analysis explored the output of five covariates in 22 studies in Ethiopia. Accordingly, the study found that distance to the market significantly affected technology adoption by less than a 5 % probability level. The regression coefficient estimate for distance to the market is 0.101, which implies that as distance to the nearest market (in kilometers) increases, farmers’ probability of adopting agricultural technology decreases by 10.1 % in Ethiopia. Rural farmers estimate that they would not be able to buy agricultural inputs and sell their output on time and at the appropriate price as the distance to the local market increases, all things remain constant. The study results are in line with the findings of [45,47].

The study also indicated that household's experience of using microcredit is positively related to agricultural technology adoption and is significant at less than a 1 % probability level (Table 3). The coefficient value of the log odds ratio was 0.1307, indicating that as household experience increased by several years, agricultural technology adoption increased by 13.07 %, ceteris paribus. This result is consistent with [4,7].

Furthermore, the annual income of a farmer positively and significantly affects the technology adoption decision at a p-value of less than a 5 % significance level. The log odds-ratio value indicates that annual income positively helps to adopt the new agricultural technology adoption by 4.3 %. This indicates that as farmers’ income increases (in thousands of Ethiopian Birr), farmers become more willing and able to adopt new agricultural technologies because they expect that income creates an excellent opportunity to buy improved inputs and fertilizers, all things remain constant. This finding is in line with the findings of [10,79].

## 5 Limitations and future research directions

The application of meta-analysis offers a robust approach for computing effect sizes by comparing two-group continuous and binary outcomes. In the context of agricultural technology adoption, this study primarily focuses on two-group comparison binary outcomes to analyze the heterogeneous effect of microcredit access. While the study incorporated factors other than microcredit that affect agricultural technology adoption through meta-regression, it is essential to note that certain factors, such as environmental and social considerations, were not incorporated into the analysis. Including those factors, specifically in the context of two-group comparisons of continuous outcome studies would enhance the depth of future agricultural technology adoption research. Moreover, the study is limited to Ethiopia and suggests a wider study area outside of Ethiopia, such as in Sub-Saharan African countries and the continental context.

## 6 Conclusion and policy remarks

This study assessed the heterogeneity status of microcredit access on agricultural technology adoption and its effect on farmers in Ethiopia. The study reviewed different agricultural adoption studies, by categorizing them into typologies such as improved seed varieties, chemical and organic fertilizers, improved livestock technologies, improved irrigation technologies, and multiple agricultural technologies. The outcome indicated that access to credit had a heterogeneous effect on Ethiopia's adoption performance of agricultural technology. Our findings indicate that the agricultural technologies adoption status of smallholder farmers is heterogeneous and low. This is mainly because of the lack of financial support schemes for smallholder farmers and other challenges.

The meta-analysis revealed that the association between access to microcredit and adoption of agricultural technology was represented by an overall odds ratio of 1.59, indicating that microcredit access has a strong effect on technology adoption among farmers in Ethiopia. The study also identified variations in microcredit access among farmers associated with agricultural product quantity and quality, credit access status, and variations of technology adoption within farmers.

The subgroup analysis of the agricultural technologies adoption studies indicated high heterogeneity in terms of improved livestock technology adoption, followed by fertilizer adoption technologies and improved seed varieties. Similarly, the heterogeneity of multiple agricultural technologies and improved irrigation technologies was moderate among adopters compared with non-adopters. Moreover, the meta-regression analysis results showed that distance to the market, household's microcredit use experience with agricultural technology adoption, and income are the moderators that affect the technology adoption decisions of Ethiopian smallholder farmers.

To promote agricultural technology adoption in Ethiopia, providing financial option information to low-level implementers such as farmers, development agents, lower-level leaders, and researchers on how to embrace new agricultural technologies in various agricultural technology categories is essential. In addition, the participation of commercial banks, microfinance institutions, and private and non-governmental organizations has a vital role in facilitating agricultural microcredit access services and other extension packages for smallholder farmers to enhance the adoption of agricultural technologies.

## Ethical approval

Ethical approval is not necessary because we utilized secondary data studies.

## Consent for publication

We agree to publish the manuscript in this journal.

## Funding

Not available.

## Data availability statement

Data will be made available upon request.

## CRediT authorship contribution statement

**Berhanu Kuma Shano:** Writing – review & editing, Visualization, Validation, Supervision, Methodology, Formal analysis, Conceptualization. **Samuel Semma Waje:** Writing – original draft, Visualization, Validation, Software, Resources, Methodology, Investigation, Formal analysis, Data curation, Conceptualization.

## Declaration of competing interest

The authors declare that they have no known competing financial interests or personal relationships that could have appeared to influence the work in this paper.
